# Characterization of *Chlorella sorokiniana* growth properties in monosaccharide-supplemented batch culture

**DOI:** 10.1371/journal.pone.0199873

**Published:** 2018-07-03

**Authors:** Shuaijie Chai, Jianan Shi, Teng Huang, Yalu Guo, Jian Wei, Meicen Guo, Liyun Li, Shijuan Dou, Lijuan Liu, Guozhen Liu

**Affiliations:** Institute of Bioenergy, Hebei Agricultural University, Baoding, Hebei Province, China; The Education University of Hong Kong, HONG KONG

## Abstract

To reveal growth properties of *Chlorella sorokiniana* UTEX 1230, four monosaccharides (glucose, fructose, galactose and xylose) were individually supplemented into medium as carbon sources for the cultivation of *C*. *sorokiniana* UTEX 1230. Supplementation with glucose increased OD_750_, biomass and lipid yield but decreased protein abundance per unit dry weight of biomass under all concentrations examined, the maximum OD_750_, biomass and lipid yield increased 2.04, 6.78 and 12.43 times, respectively, compared with autotrophic controls. A low concentration of glucose (<4 g/L) simultaneously promoted the biosynthesis of chlorophylls and protein abundance per unit culture volume, but decreased the lipid content per unit dry weight of biomass and all supplemented glucose can be exhausted within 7 days. Higher glucose concentrations (≥4 g/L) decreased the biosynthesis of chlorophylls and protein abundance per unit culture volume, but increased the lipid content per unit dry weight of biomass. In glucose supplemented scenario, *C*. *sorokiniana* UTEX 1230 growth was light-independent. Supplementation with fructose promoted *C*. *sorokiniana* UTEX 1230 growth to a much lesser extent compared with glucose, whereas supplementation with galactose had no effect and supplementation with xylose even inhibited growth. Our findings represent basic experimental data on the effect of monosaccharides and can serve as the basis for a robust cultivation system to increase biomass and lipid yield.

## Introduction

Because large-scale use of fossil fuels has caused severe environmental pollution and is leading to their depletion, the development and use of renewable and sustainable energy sources is needed. In recent years, bioenergy has emerged as an attractive option because it can be produced from a variety of feedstocks, including vegetable oils, seed oils and other lipid-rich biomass materials [1–3]. Large-scale cultivation of oil crops is limited, however, by low oil yields and competition with agricultural land. Microalgae are theoretically more promising bioenergy resources offering significant advantages, such as fast growth [4], short growth cycles, high lipid yields [5], bioactive compounds production [6] and the ability to grow on non-crop lands and in salt water [7–11] or waste water [12]. While primary technologies involving microalgae cultivation and lipid extraction have already been established [13], low yields and high bioenergy costs are key bottlenecks to industrial-scale exploitation of microalgae. Overcoming these challenges will require major efforts to optimize cultivation systems and further elucidate the mechanisms underlying lipid biosynthesis.

Although microalgae are typically cultivated under photoautotrophic conditions, light transmittance is attenuated dramatically as cell density increases, which results in extra costs for light supplementation. The use of exogenous carbon sources with either heterotrophic (without light) or mixotrophic (with light) culture modes have been reported to increase the biomass of many kinds of microalgae. For example, Bhatnagar et al. (2011) compared the biomass of three species of microalgae (*Chlamydomonas globosa*, *Chlorella minutissima* and *Scenedesmus bijuga*) and found 3 to 10 times increased biomass accumulation when they were cultivated mixotrophically as compared with autotrophic culture [14]. The biomass, growth rate and lipid content of *Chlorella* strains can be raised by supplementation of the culture medium with organic carbon sources [15]. Nitrogen depletion, one of the most common methods to induce lipid biosynthesis in microalgal cells, is not amenable to industrial use because of its high costs and negative effects on biomass production [16].

*Chlorella sorokiniana* is one of the most commonly used chlorella strain for mass cultivation [17]. *Chlorella sorokiniana* UTEX 1230 was reported for the first time in 1953 [18], and was identified as an ideal candidate for mixotrophic cultivation that offers great potential in the production of renewable biomass for bioenergy applications [19]. Cultivation of UTEX 1230 in medium supplemented with 0.1 M glucose has been observed to yield 0.56 g biomass/g glucose in an outdoor enclosed photobioreactor and 0.35 g biomass/g glucose under dark conditions [20]. Kobayashi et al. (2013) have reported that UTEX 1230 produces higher biomass, proteins and starch with less lipid accumulation compared with two other strains (*Chlorella sorokiniana* CS-01 and UTEX 2714) [21]. Among 30 *Chlorella* strains, UTEX 1230 under heterotrophic conditions has been found to grow five times faster than under autotrophic conditions, with a doubling of biomass in 9 h and a maximum dry weight lipid content of 39% [22]. UTEX 1230 has also been used as a model to verify the effectiveness of brefeldin A, a candidate lipid inducer identified from a screening of 1,717 types of chemicals [23]. Because of this abundant background information and its superior characteristics, UTEX 1230 was used in this study to investigate the influence of monosaccharides on *Chlorella* strains.

Various organic carbon sources, including polysaccharides, disaccharides, monosaccharides, starch and ammonium acetate, have been used for microalgal cultivation, but systematic comparison data between primary monosaccharides are limited. To develop a cultivation mode that stably enhances biomass production and lipid yield and to reveal the influences of different monosaccharides on microalgal cultivation properties, *C*. *sorokiniana* UTEX 1230 was cultivated in Bold’s Basal Media (BBM) supplemented with either glucose, fructose, galactose or xylose in combination with light/dark treatments. Following these treatments, biomass, lipid content, lipid yield, and chlorophyll and total protein abundances were investigated and compared.

## Materials and methods

### Experimental materials

*Chlorella sorokiniana* UTEX 1230 was purchased from the Culture Collection of Algae at the University of Texas at Austin (UTEX).

### Methods

#### Culture conditions for *Chlorella sorokiniana* UTEX 1230

*C*. *sorokiniana* UTEX 1230 cells (1 ml) was inoculated in 30 ml medium and grown either autotrophically, heterotrophically or mixotrophically in BBM at 25±1 °C with shaking at 130 rpm under 100 μmol/m^2^·s white fluorescent lamp illumination and a 14/10-h light/dark photoperiod. The initial OD_750_ was about 0.15. The BBM was prepared as reported [15]. Solid culture medium contained 15 g agar powder per liter of medium, which was autoclaved at 121 °C for 25 min. Monosaccharide stock solutions were 0.5 g/mL (glucose) or 0.1 g/mL (fructose, galactose and xylose), prepared by filtering through 0.2 micron filter, and then supplemented to autoclaved medium. For heterotrophic culture, Erlenmeyer flasks containing 30 mL medium were completely covered with aluminum foil.

#### Measurement of chlorophyll in *C*. *sorokiniana* UTEX 1230 cells

Chlorophyll was extracted from *C*. *sorokiniana* UTEX 1230 with DMSO (Tianjin Kemiou Chemical Reagent Co., China) and measured [24]. Two milliliters of each culture was added to a 2-mL tube and centrifuged at 7,378 g for 3 min. After discarding the supernatant, the precipitate was re-suspended in 2 mL DMSO (preheated to 60 °C) by vortexing for 10 min, followed by centrifugation at 7,378 g for 3 min. The supernatant (chlorophyll extract) was transferred to another Eppendorf tube and diluted with DMSO to an appropriate concentration. Optical densities at 480, 649 and 665 nm were measured with a spectrophotometer (7200 Unico, Shanghai, China) and chlorophyll concentrations were calculated using the following formulas:

$$\mathrm{Chlorophyll}\;a\;(\mathrm{ChlA})\;(\mathrm{mg}/L)=12.47\;({OD}_{665})-3.62\;({OD}_{649})$$ (1)

$$\mathrm{Chlorophyll}\;b\;(\mathrm{ChlB})\;(\mathrm{mg}/L)=25.06\;({OD}_{649})-6.5\;({OD}_{665})$$ (2)

$$\mathrm{Total}\;\mathrm{carotenoid}\;(\mathrm{mg}/L)=[1000\;({OD}_{480})-1.29\;(\mathrm{ChlA})-53.78\;(\mathrm{ChlB})]/220$$ (3)

Totalchlorophyll(mg/L)=(1)+(2)+(3) (4)

The experiments were performed in triplicate.

#### Cell number counts and cell morphological observations

A droplet of 7-day culture was dripped onto a hemocytometer to observe cell morphology and count cells under an optical microscope (Olympus BH-2, Japan). Each sample was measured three times and the average was used to calculate the number of *C*. *sorokiniana* UTEX 1230 cells per milliliter.

### Biomass measurement

To measure biomass, 15 mL of *C*. *sorokiniana* UTEX 1230 culture was added to a pre-weighed (W1) Eppendorf tube and centrifuged at 5,000 *g* for 5 min. The precipitate was dried at 80 °C to a constant weight in an electronic oven. Biomass (W; g/L) was calculated as follows:

W=(W2−W1)×1000/V (5)

Where W1 is the weight of the tube (g), W2 is the weight of dried microalgae and the tube, and V is the culture volume. The experiments were performed in triplicate.

#### Measurement of *C*. *sorokiniana* UTEX 1230 cell density

To determine cell density, the optical density of 3 mL of *C*. *sorokiniana* UTEX 1230 culture was measured at 750 nm with a spectrophotometer (7200 Unico). Individual cultures were measured in triplicate.

#### Measurement of lipid content

The sulfo-phospho-vanillin (SPV) method was used to determine the lipid content of *C*. *sorokiniana* UTEX 1230 as reported previously [25, 26]. The OD_530_ of the culture was measured with a spectrophotometer (7200 Unico). For construction of a standard curve, a stock solution was prepared by dissolving 100 mg triolein in 100 mL chloroform. After 0, 10, 20, 30, 40, 50, 60, 70 and 80 μL aliquots of stock solution were blown dry in a fume hood, 100 μL of distilled water and 1 mL of sulfuric acid were added to the residues and mixed by pipetting. The mixtures were incubated in a 100 °C water bath for 10 min and then allowed to cool to room temperature before adding 5 mL of phosphovanillin. Phosphovanillin reagent was prepared by dissolving 0.6g vanillin in 100 ml ethanol (90%, v/v) and stirred continuously. Subsequently 400 ml of concentrated phosphoric acid was added to the mixture, and the resulting reagent was stored in the dark until use. After brief shaking, the tubes were incubated with shaking in a 37 °C water bath for 15 min. A standard curve was drawn based on OD_530_ values and triolein concentrations. To determine the lipid concentration of *C*. *sorokiniana* UTEX 1230 cells, 2 mL of each culture was centrifuged at 5000 *g* for 5 min and the supernatant was discarded. Precipitates were washed twice with distilled water and re-suspended in 100 μL distilled water and 1 mL of sulfuric acid were added to the residues. The mixtures were incubated in a 100 °C water bath for 10 min and then allowed to cool to room temperature before adding 5 mL of phosphovanillin. OD_530_ were measured as described above and lipid contents were calculated based on the standard curve. All reagents were purchased from Sangon Biotech Co., Ltd. (Shanghai, China). The experiments were performed in triplicate.

#### Measurement of monosaccharide concentration in the culture medium

The concentration of monosaccharide in the culture medium was determined by the 3, 5-dinitrosalicylic acid method in triplicate [27]. Standard curves were drawn for glucose, fructose, galactose and xylose. After centrifuging 1 mL of culture at 5000 *g* for 5 min, 0.5 mL of the supernatant was used to measure monosaccharide concentration according to the standard curves for each of the four monosaccharides. The difference between initial and final monosaccharide concentration was calculated and used as the amount of monosaccharide in the culture. In addition, the biomass increase resulting from glucose supplementation (Bi) was calculated from the following equation:

$$\mathrm{Bi}=(B_{h}-\;B_{d})/(C_{0}-\;C_{n})$$ (6)

where B_h_ is biomass under heterotrophic conditions, B_d_ is biomass under dark conditions (without glucose), C_0_ is initial glucose concentration and C_n_ is residual glucose concentration.

#### Total protein extraction

Approximately 20 mL of *C*. *sorokiniana* UTEX 1230 culture was harvested and centrifuged at 5000 g for 5 min. After discarding the supernatant, 1 mL of Laemmli sample buffer [62.5 mM Tris-HCl (pH6.8), 2% sodium dodecyl sulfate (SDS), 5% (v/v) β-mercaptoethanol, 10% (v/v) glycerol and 0.01% bromophenol blue] [28] was added to the pellet. The solution was vortexed for 10 s and then cooled on ice for 20 s, repeats the process for 10 to 15 times and centrifuged at 15,000 *g* for 15 min. The supernatant was collected as total protein. The protein samples were mixed with loading buffer [250 mM Tris-HCl (pH6.8), 10% SDS (w/v), 0.5% bromophenol blue (w/v), 50% glycerol (v/v) and 5% β-mercaptoethanol (w/v)], boiled for 10 min and stored at −20 °C until use.

#### Total protein quantification

Two parallel methods were used to determine total protein abundances: Coomassie brilliant blue (CBB) R-250 gel staining and the Bradford method [29]. For CBB staining, total proteins were separated by sodium dodecyl sulfate-polyacrylamide gel electrophoresis (SDS-PAGE), stained with CBB, de-stained and photographed. ImageJ software [30] was used to calculate the signal intensity of the bands from the CBB results. The intensity of each lane was measured three times and the average was calculated. Relative protein abundance per unit culture volume was calculated by dividing the intensity with loading culture volume. Relative protein abundance per unit dry weight of biomass was calculated by dividing the intensity with corresponding biomass.

## Results

### Morphological alterations of *C*. *sorokiniana* UTEX 1230 grown in monosaccharide-supplemented medium

**Fig 1:**
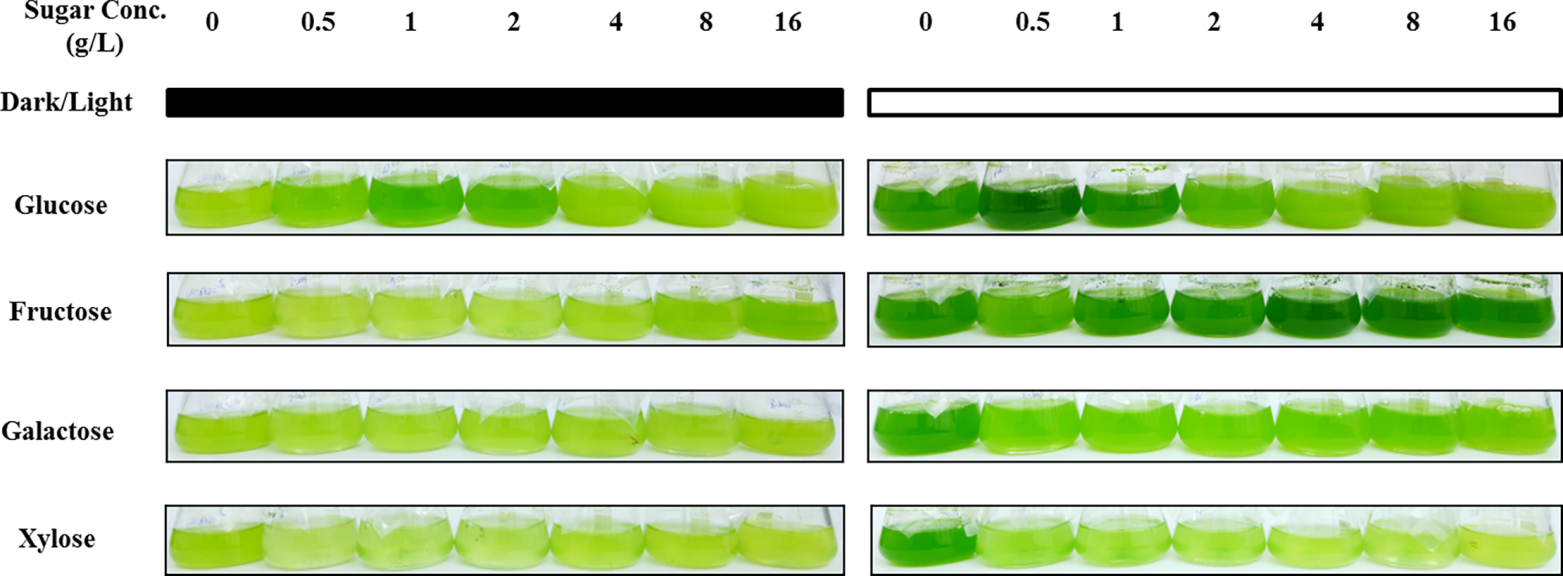
Morphological appearance of *Chlorella sorokiniana* UTEX 1230 grown in monosaccharide-supplemented medium. Photos of *C*. *sorokiniana* UTEX 1230 cultivated for 7 days in medium supplemented with different concentrations of monosaccharides. Left (black bar) and right (white bar) panels show flasks cultured under dark and light conditions, respectively. The photos were taken with a digital camera against a white background.

To evaluate the influence of monosaccharides on *C*. *sorokiniana* growth, UTEX 1230 was grown in BBM supplemented with various concentrations of four different monosaccharides (glucose, fructose, galactose or xylose) in the presence or absence of light. The photographs in Fig 1 show the morphology of *C*. *sorokiniana* UTEX 1230 cultured for 7 days under various monosaccharide concentrations. As can be seen in the figure, cultures grown in the absence of supplemental monosaccharide were typically yellow when incubated in darkness. After supplementation with low concentrations of glucose (0.5, 1 or 2 g/L), the cultures turned green, while the addition of higher glucose concentrations (4, 8 or 16 g/L) resulted in a light yellow appearance. High concentrations of fructose led to a green-colored culture. Supplementation with galactose or xylose lightened the color of the culture; this was especially true for xylose-treated cultures. Under light conditions in the absence of monosaccharide, the cultures displayed the typical green color. Supplementation with a low concentration of glucose (0.5 or 1 g/L) turned the culture deep green compared with the control, while higher concentrations (2, 4, 8 or 16 g/L) of glucose resulted in a yellow color. No visible color difference was observed between fructose-treated cultures and the control. Galactose or xylose supplementation caused yellowing of the culture, with xylose-treated cultures a lighter yellow than galactose-treated ones.

To detect possible morphological alterations at cellular level, cells of *C*. *sorokiniana* UTEX 1230 were observed under a microscope 7 days after supplementation with monosaccharides. Although no size alteration was observed between *monosaccharide* -treated cultures and controls, many cells displayed a compact and dense morphology in glucose-supplemented medium, which may have been due to an increase in the contents of cells. However, supplementation with fructose, galactose or xylose led to no significant differences compared with the controls (S1 Fig).

### Pigment alterations of *C*. *sorokiniana* UTEX 1230 grown in monosaccharides-supplemented mediums

**Fig 2:**
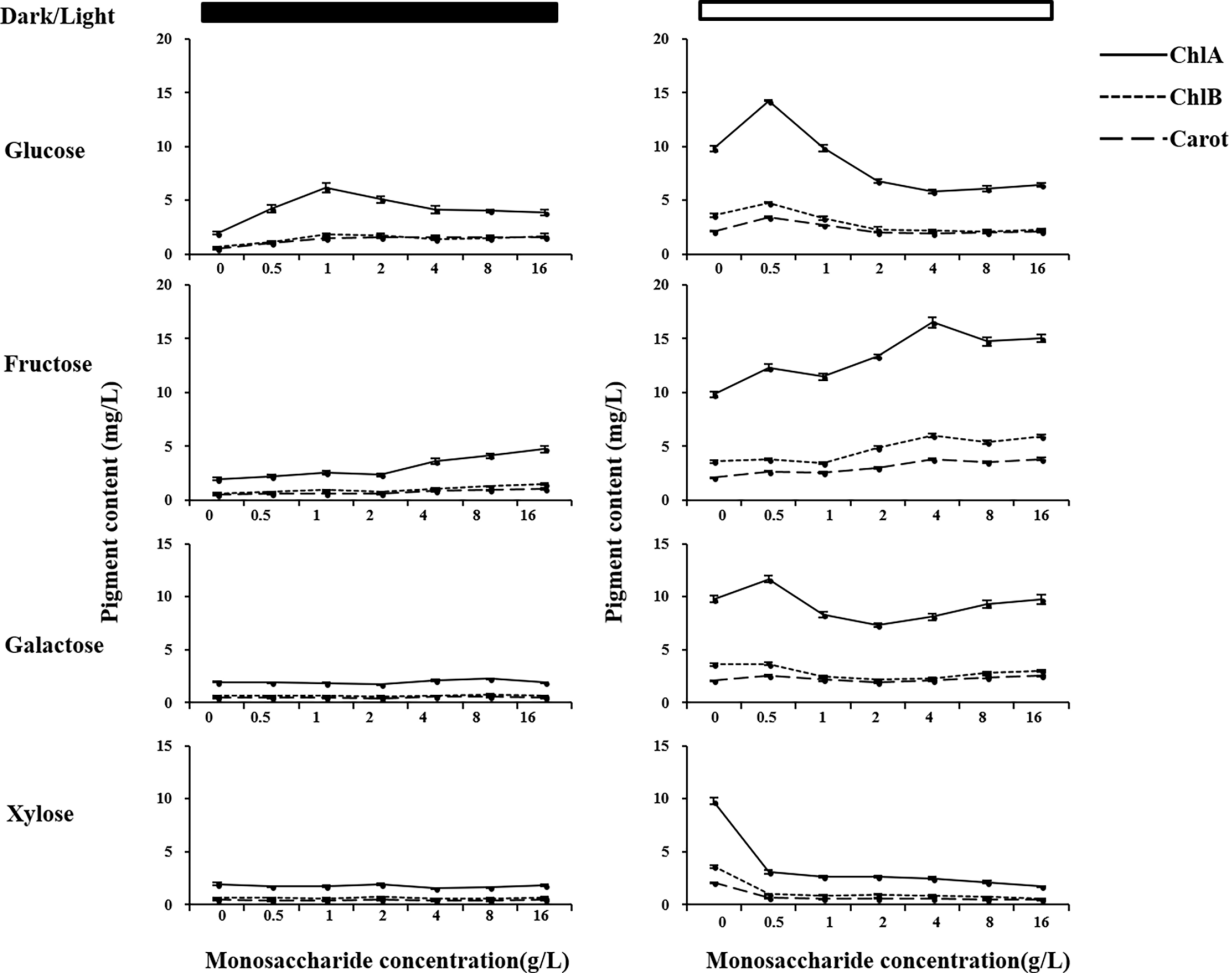
Pigment contents of *Chlorella*. *sorokiniana* UTEX 1230 grown in monosaccharides-supplemented mediums. The contents of DMSO extracted pigment from *C*. *sorokiniana* UTEX 1230 were determined by the optical density under corresponding wavelength. The line graph was drawn, x- and y-axes on the graph correspond to monosaccharide concentration and pigment contents, respectively. The left panel (black bar) showed the pigment extracted from cultures under dark condition. The right panel (blank bar) showed the pigment extracted from cultures under light condition. Data shown as mean +/-SD, n = 3. ChlA: chlorophyll a; ChlB: chlorophyll b; Carot: total carotenoid.

To determine why these color alterations occurred during *C*. *sorokiniana* UTEX 1230 growth, chlorophyll a, b and total carotenoids were extracted with DMSO and their contents were spectrometrically measured at the corresponding wavelengths (Fig 2). As shown in Fig 2, pigment contents of *C*. *sorokiniana* UTEX 1230 cultivated in light were generally higher than those cultured in darkness. Supplementation with 1 g/L glucose resulted in the highest pigment contents under light conditions, whereas 0.5 g/L glucose led to the highest pigment contents in darkness. Higher glucose concentrations decreased pigment contents. A clear correlation was observed between chlorophyll content and the color of the culture (Fig 1), indicating that the color of the culture was dependent on pigment synthesis. Low glucose concentrations were able to promote synthesis of pigments, whereas high glucose concentrations had an inhibitory effect on pigment synthesis. Fructose supplementation increased the chlorophyll a contents for about 50%, while the addition of galactose caused a subtle change in pigment contents. Supplementation with xylose, even at the lowest concentration, completely inhibited pigment synthesis, thereby causing pigment contents of *C*. *sorokiniana* UTEX 1230 cultured under light conditions to be equivalent to those cultured in darkness.

### Cell number and density alterations of *C*. *sorokiniana* UTEX 1230 grown in monosaccharide-supplemented medium

**Fig 3:**
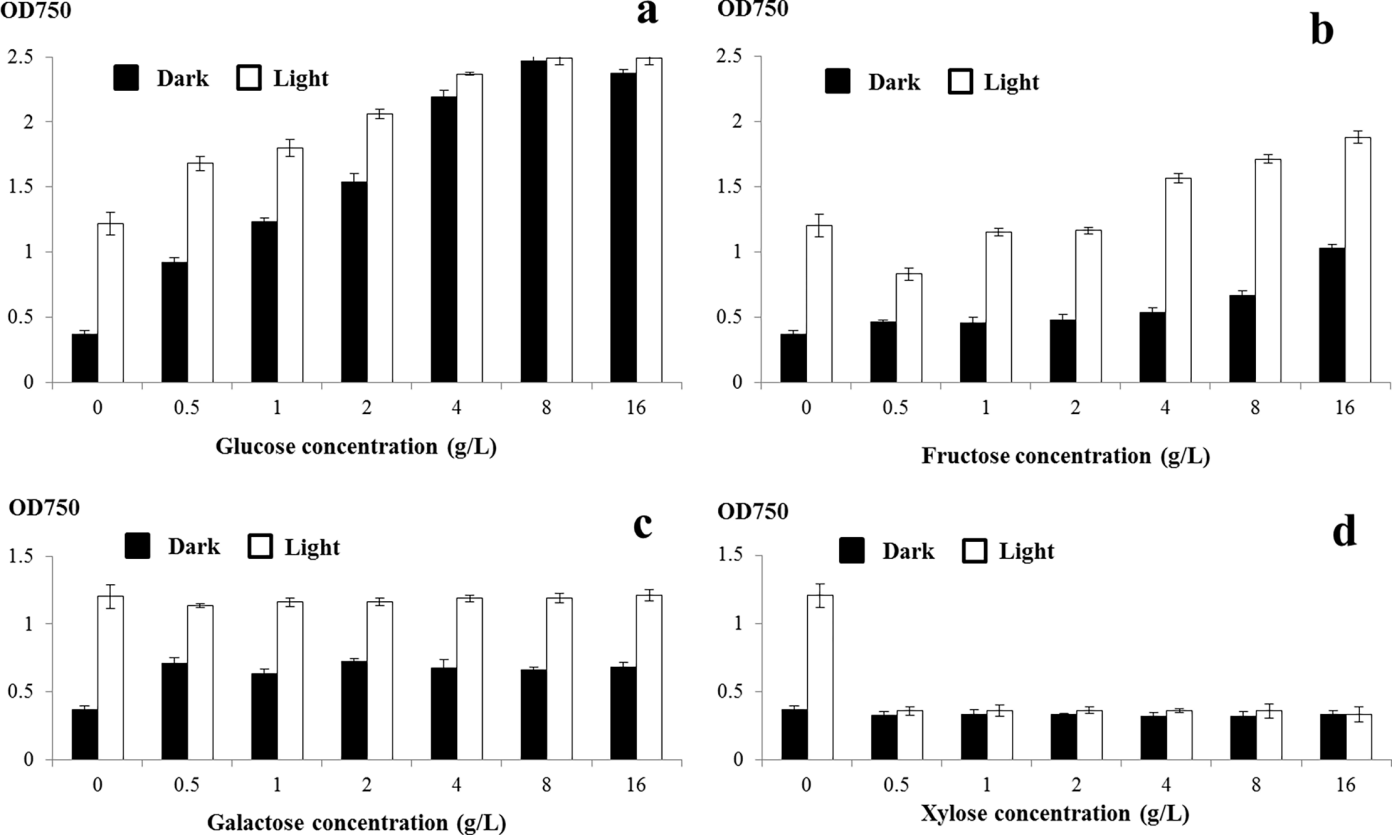
Cell density alterations to *Chlorella sorokiniana* UTEX 1230 grown in monosaccharide-supplemented medium. The density of *C*. *sorokiniana* UTEX 1230 cells collected after 7 days culture in monosaccharide-supplemented medium were determined by spectrophotometry at 750 nm. a, b, c, and d show cell densities following culture in glucose-, fructose-, galactose- and xylose-supplemented media, respectively. Data shown as mean +/-SD, n = 3. Black bar: dark conditions; white bar: light conditions.

To monitor algal growth quantitatively, cell density was measured with a spectrophotometer at 750 nm and cell numbers were counted using a hemocytometer. Alterations to OD_750_ caused by supplementation with monosaccharides are shown in Fig 3. In general, OD_750_ correlated with counted cell numbers S2 Fig and S3 Fig. Under dark conditions, supplementation with low levels of glucose (0.5–4 g/L) promoted a proportional increase in OD_750_, with saturation observed when high levels of glucose (8–16 g/L) were added. OD_750_ saturation also occurred under light conditions. At lower glucose concentrations, cell densities obtained under light conditions were higher than those generated under darkness, which demonstrates that both light and glucose contribute to algal growth and play cumulative effect. Under higher glucose concentrations, OD_750_ saturation levels were identical under both light and dark conditions; they were 2.04 times as high as those of the photoautotrophic control, which suggests that *C*. *sorokiniana* UTEX 1230 growth is light-independent in the presence of sufficient added glucose (Fig 3A). OD_750_ values of cultures supplemented with fructose are shown in Fig 3B. Low fructose concentrations had no effect on algal growth, whereas higher fructose concentrations were able to increase the OD_750_ slightly—but much less than that supplementation with glucose. No effect was observed in galactose-supplemented treatments at tested concentrations, which indicates that galactose had no impact on the growth of *C*. *sorokiniana* UTEX 1230 (Fig 3C). Supplementation with xylose had no effect on *C*. *sorokiniana* UTEX 1230 grown in darkness, whereas xylose significantly inhibited algal growth under light conditions (Fig 3D).

### Biomass analysis of *C*. *sorokiniana* UTEX 1230 in monosaccharide-supplemented medium

**Fig 4:**
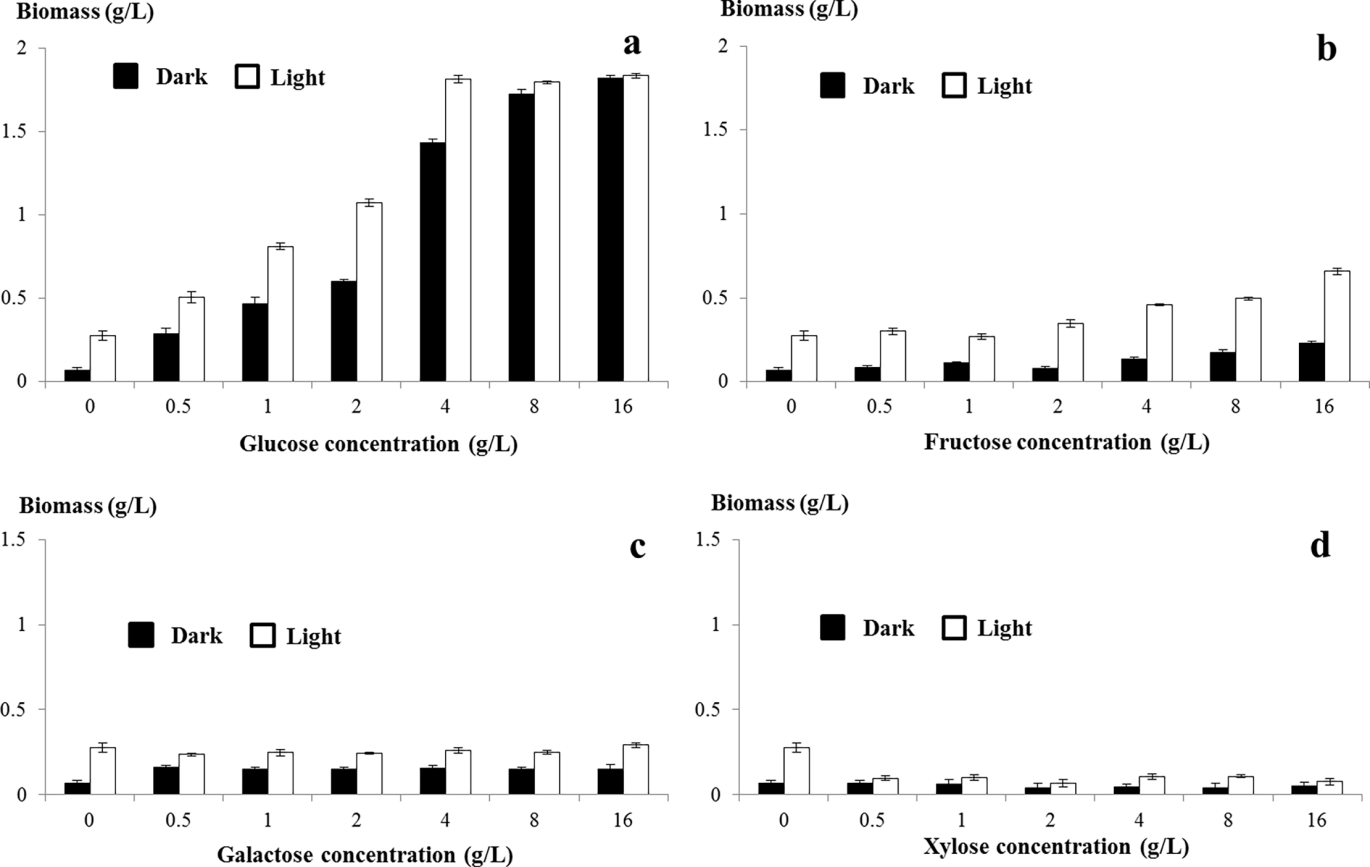
Biomass analysis of *Chlorella sorokiniana* UTEX 1230 in monosaccharide-supplemented medium. *C*. *sorokiniana* UTEX 1230 cells were collected after 7 days and dried to a constant weight at 80 °C to weigh biomass. X- and y-axes on the graph correspond to monosaccharide concentration and biomass (g/L), respectively. a, b, c and d show the biomass of cells cultivated in glucose-, fructose-, galactose- and xylose-supplemented media, respectively. Data shown as mean +/-SD, n = 3. Black bar: dark conditions; white bar: light conditions.

To analyze biomass, *C*. *sorokiniana* UTEX 1230 was collected by centrifugation and dried to a constant weight. As shown in Fig 4, *C*. *sorokiniana* UTEX 1230 biomass increased along with increasing glucose concentration (Fig 4A). Under both dark (heterotrophic) and light (mixotrophic) conditions, biomass in the 16 g/L glucose treatment was as high as 1.833 g/L (6.54 times higher than control levels). As the fructose concentration was increased, the biomass of *C*. *sorokiniana* UTEX 1230 under dark conditions increased slightly. While under light conditions, the biomass was 2.32 times higher than the control—a much smaller impact than that resulting from supplementation with glucose (Fig 4B). No effect on *C*. *sorokiniana* UTEX 1230 biomass was observed in galactose-treated medium (Fig 4C). Supplementation with xylose resulted in a decrease in biomass, which indicates that xylose can inhibit the growth of *C*. *sorokiniana* UTEX 1230 (Fig 4D).

### Lipid yield analysis of *C*. *sorokiniana* UTEX 1230 in monosaccharide-supplemented medium

**Fig 5:**
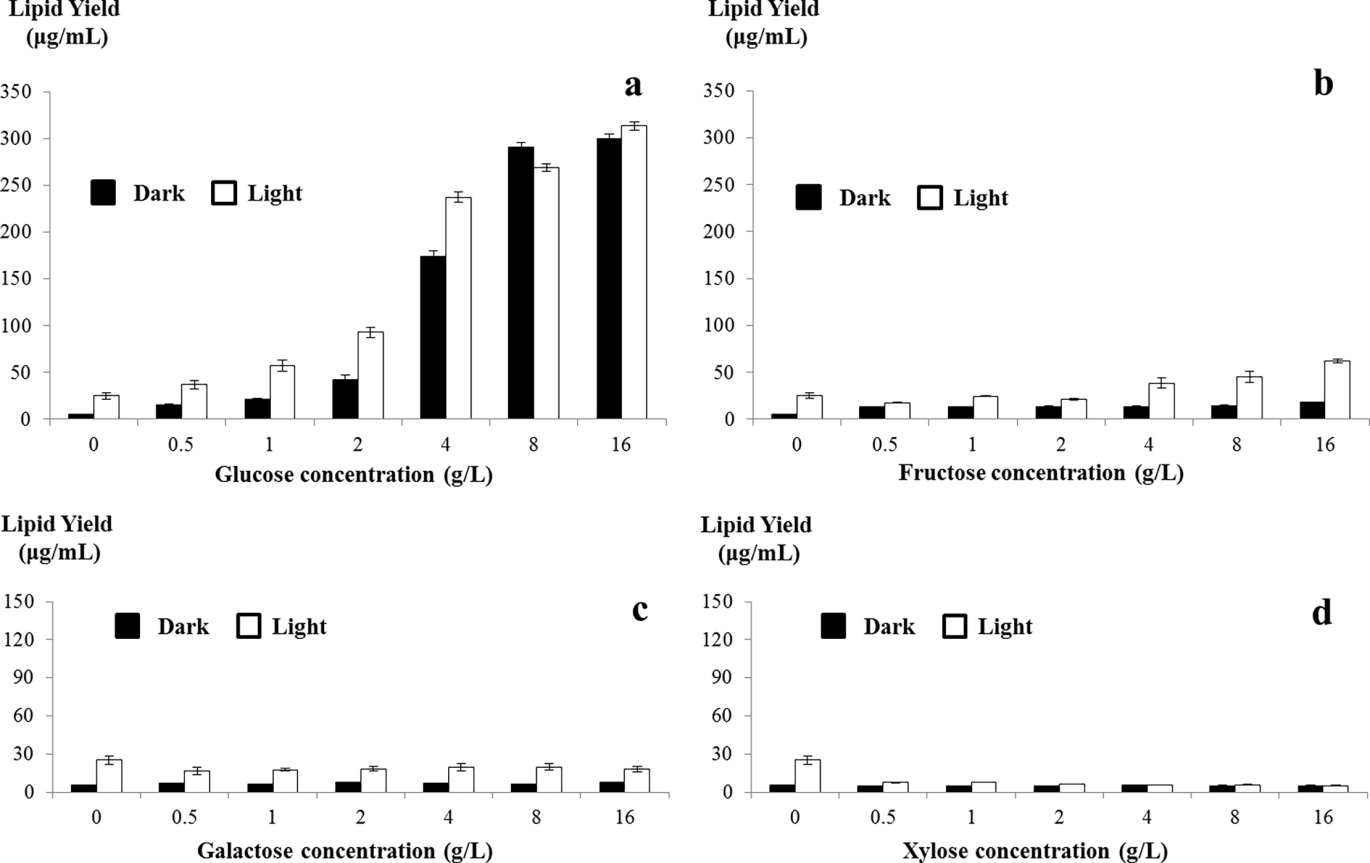
Lipid yield of *Chlorella sorokiniana* UTEX 1230 in monosaccharide-supplemented medium. Lipid yield of *C*. *sorokiniana* UTEX 1230 cells collected from monosaccharide-supplemented medium after 7 days culture was determined by the sulfo-phospho-vanillin method. Lipid yield per mL of culture was calculated. X- and y-axes on the graph correspond to monosaccharide concentration and lipid yield (μg/mL), respectively. a, b, c and d show the lipid yield of cells cultivated in glucose-, fructose-, galactose- and xylose-supplemented media, respectively. Data shown as mean +/-SD, n = 3. Black bar: dark conditions; white bar: light conditions.

To quantify lipids in cells, algal cultures were collected by centrifugation, washed twice with distilled water to eliminate possible interference due to residual monosaccharide, and analyzed for lipid yield by the SPV method. Lipid yield per volume of medium is shown in Fig 5.

As can be seen in Fig 5A, total lipid yield increased rapidly along with increasing glucose up to 4 g/L. At concentrations of 0.5, 1, 2 and 4 g/L, cells cultivated in light produced more lipid than cells in darkness, indicating that light can contribute to lipid biosynthesis. Under higher concentrations of glucose (8 and 16 g/L), total lipid yield was saturated and independent of light conditions. At this point, the cultures produced 12.43 times more lipid than the control. Supplementation with fructose resulted in a slight increase in lipid yield, but the final yield was far below the yield in glucose-supplemented *C*. *sorokiniana* UTEX 1230 cells (Fig 5B). No effect on lipid yield was observed in galactose-supplemented culture (Fig 5C). In cells cultivated with xylose under light conditions, lipid yield even decreased (Fig 5D).

Lipid content per unit dry weight of biomass (μg/mg) was also calculated. These measurements revealed that low glucose concentrations led to lipid contents lower than the control, whereas higher concentrations were responsible for a 1.82-fold increase in lipid content relative to the control (S4 Fig). Supplementation with other monosaccharides did not alter lipid content significantly. Overall, supplementation with glucose (16 g/L) increased *C*. *sorokiniana* UTEX 1230 biomass and lipid content relative to the control by 6.78 times and 1.82 times, respectively. In theory, lipid yield can be increased to 12.34 (6.78 × 1.82) times than that of control, while it was actually increased 12.43 times (Fig 5). The reference to theory and actual lipid yields were perfectly match for each other.

### Consumption of monosaccharides during *C*. *sorokiniana* UTEX 1230 cultivation

**Fig 6:**
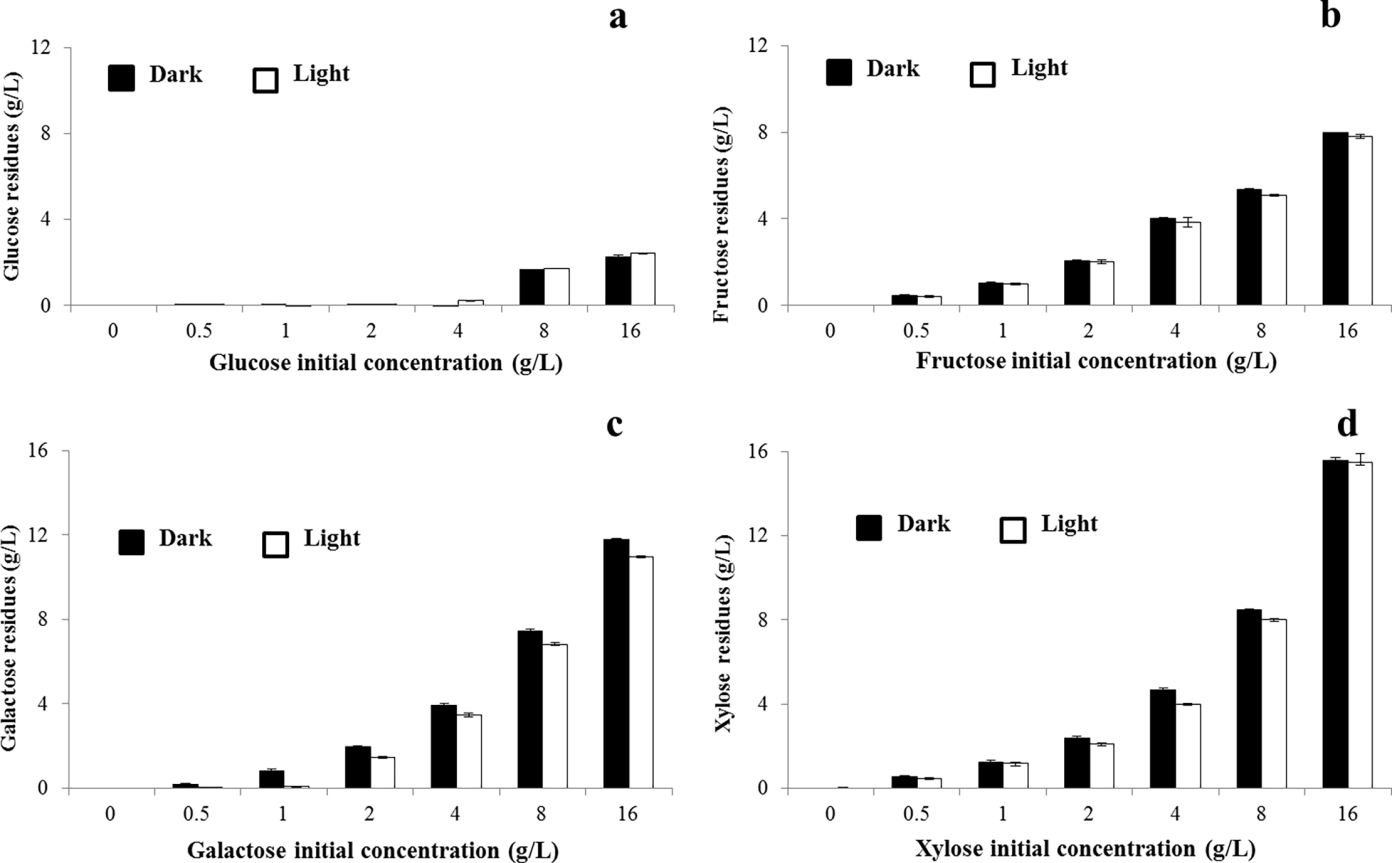
Residual monosaccharide after 7 days cultivation of *Chlorella sorokiniana* UTEX 1230. Residual monosaccharides (final concentration at 7 days) as measured by the dinitrosalicylic acid method and calculated based on a standard curve. a, b, c and d show remaining monosaccharide from glucose-, fructose-, galactose- and xylose-supplemented media, respectively. Data shown as mean +/-SD, n = 3. Black bar: dark conditions; white bar: light conditions.

To analyze monosaccharide consumption during *C*. *sorokiniana* UTEX 1230 cultivation, residual monosaccharide in the culture medium was measured after 7 days cultivation (Fig 6). At initial concentrations below 4 g/L, glucose was exhausted within 7 days. When the initial concentration of glucose was 8 g/L, the residual glucose concentration was 1.5 g/L, while an initial concentration of 16g/L resulted in 2 g/L glucose remaining after 7 days cultivation (Fig 6A). Supplementation with 0.5 g/L glucose led to a biomass increase per unit of glucose of 0.44 g/g glucose, while 16 g/L glucose was responsible for a biomass increase per unit of glucose of 0.12 g/g glucose. No fructose was used when the initial supplied concentration was below 4g/L. At initial concentrations of 8 and 16 g/L, however, 3 and 8 g/L of fructose were respectively consumed (Fig 6B). Galactose added at a low initial concentration was barely used, while 1 and 5 g/L were consumed from initial concentrations of 8 and 16g/L, respectively (Fig 6C). All supplemental xylose remained in the culture medium after 7 days of culturing (Fig 6D).

The above-mentioned results imply that glucose can be used as a carbon source, with a fraction remaining when a high initial concentration is applied. At a sufficiently high initial concentration, some fructose and galactose can be used. Xylose cannot be used as carbon source in *C*. *sorokiniana* UTEX 1230 cultivation. On the basis of the four monosaccharides remaining and calculated consumption, a clear correlation was evident between biomass production and lipid yield. The monosaccharide most heavily used by *C*. *sorokiniana* UTEX 1230 was glucose, with accordingly the highest OD_750_, biomass and lipid yield. Fructose and galactose were less extensively used, as evidenced by less pronounced increases in OD_750_, biomass and lipids. Xylose could not be used; moreover, it inhibited the growth of *C*. *sorokiniana* UTEX 1230, with consequent decreases in biomass and lipid yield.

### Decreased protein levels of *C*. *sorokiniana* UTEX 1230 cells cultivated in glucose-supplemented medium

As revealed by the above results, supplementation with glucose dramatically promoted *C*. *sorokiniana* UTEX 1230 growth. To aid understanding of the underlying mechanism, total protein was extracted from *C*. *sorokiniana* UTEX 1230 cultivated under different glucose concentrations.

**Fig 7:**
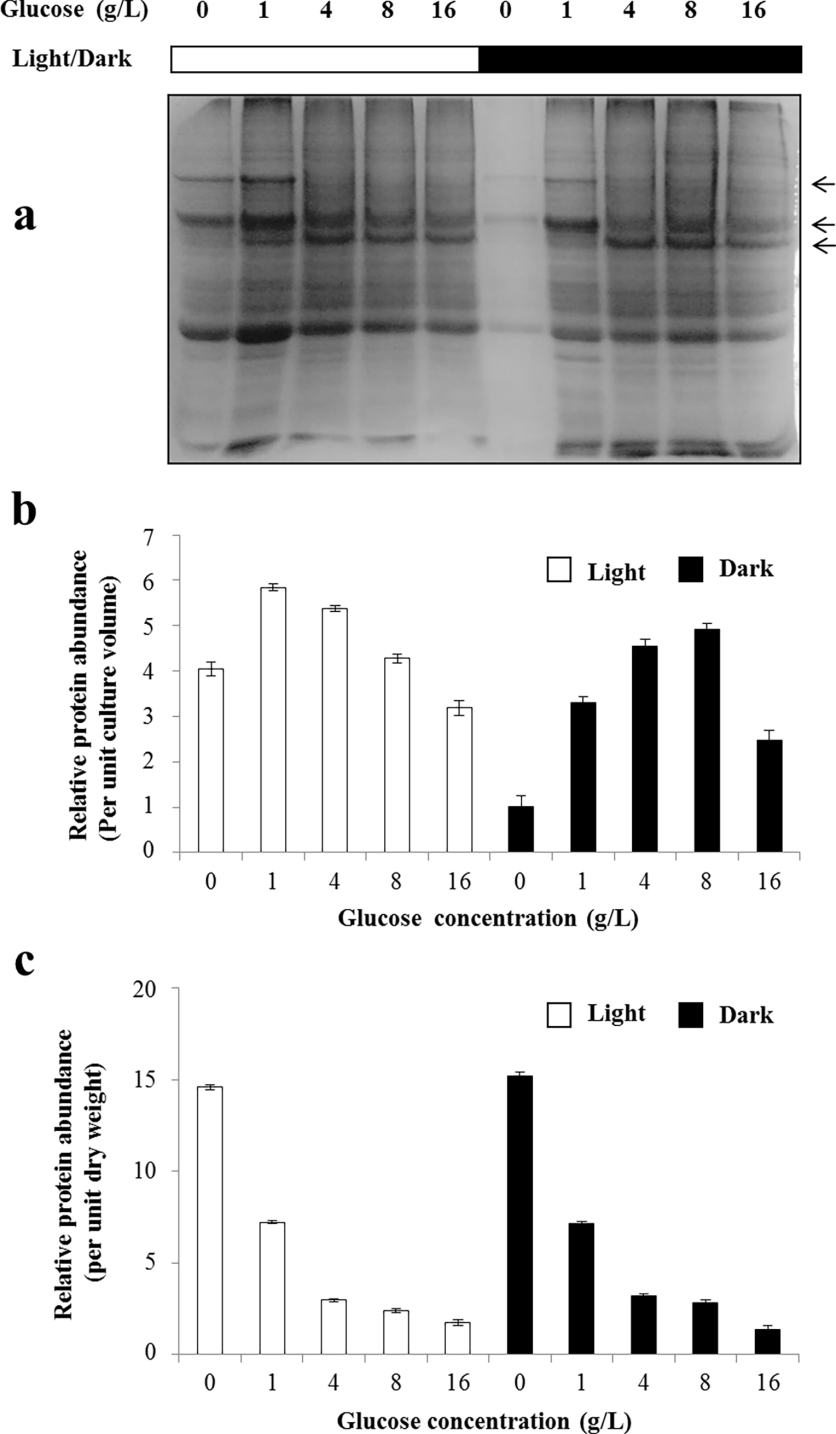
Relative protein abundances of *Chlorella sorokiniana* UTEX 1230 cells cultivated in glucose-supplemented medium. *C*. *sorokiniana* UTEX 1230 cells cultured in glucose-supplemented medium were harvested. Extracted total protein was separated by SDS-PAGE (10%) and stained with Coomassie brilliant blue (a). Arrows designated the bands with changed intensity at different glucose concentration. Signal intensities were extracted by ImageJ software, and showed as relative protein abundance per unit culture volume (b). Relative protein abundance per unit dry weight (c) was calculated by dividing with the amount of biomass. Data shown as mean +/-SD, n = 3. Black bar: dark conditions; white bar: light conditions.

Extracted total protein was separated by SDS-PAGE and stained with CBB (Fig 7A). As shown in Fig 7A, it is interesting to note that a number of up/down-regulated bands were observed on the CBB-stained gels, thus revealing glucose concentration-dependent regulation and suggesting that the expressions of at least some specific proteins were changed. The identification and functional analysis of these proteins may provide helpful clues for understanding the mechanism of glucose metabolism in *C*. *sorokiniana* UTEX 1230. Signal intensities were extracted by ImageJ software, and showed as relative protein abundance per unit culture volume (Fig 7B). It can be seen that protein abundance increased as glucose supplemented in medium. Low glucose concentrations increased biomass, thereby resulting in a slight increase in total protein abundance. In light condition, addition of 1g/L glucose resulted the highest protein abundance, while in dark condition, 8g/L glucose was needed to reach the highest protein abundance. In addition, relative protein abundance per unit dry weight of biomass was also showed in Fig 7C. It showed that protein abundance per unit dry weight of biomass decreased as glucose concentration increased: the higher the glucose concentration, the lower the abundance of total protein. At higher concentrations, supplementation with glucose proportionally decreased *C*. *sorokiniana* UTEX 1230 total protein abundance. In addition, the relative protein abundances per cell of *Chlorella sorokiniana* UTEX 1230 cultured in glucose-supplemented medium showed similar pattern (S5 Fig). In the protein to biomass ratio aspect, it is about 55% in the glucose-free medium control. While the protein to biomass ratio is only 5.5% in 16 g/L glucose-supplemented samples, 90% decrease compared with the control. Meantime, total protein was also measured by the Bradford method and found to be well correlated with the CBB results (S6 and S7 Figs).

## Discussion

Four monosaccharides (glucose, fructose, galactose and xylose) were individually supplemented into medium as carbon sources for the cultivation of *C*. *sorokiniana* UTEX 1230. It was found that supplementation with glucose promoted the growth of *C*. *sorokiniana* UTEX 1230 dramatically. Supplementation with fructose promoted *C*. *sorokiniana* UTEX 1230 growth to a lesser extent compared with glucose, whereas supplementation with galactose had no effect and supplementation with xylose inhibited growth.

### The effect of monosaccharide supplementation on the biomass of *Chlorella* species

Woodworth et al. [31] found that the biomass of *C*. *vulgaris* cultured under mixotrophy was higher than that under autotrophy at the early stage following supplementation with glucose (20g/L), whereas the biomasses of heterotrophic and mixotrophic cultures were nearly the same at the saturated stage. The authors also noticed a decrease in pigments, especially chlorophyll a, which was possibly due to the inhibitory effect of glucose on photosynthesis. The conclusion for the effect of glucose with *C*. *vulgaris* matched with our results in this report. To investigate the impact of glucose on *C*. *protothecoides*, Li et al. [32] applied a two-step process in which glucose was added in the first step and then removed in the second. Glucose was found to influence biomass, lipid, chlorophyll, protein and starch levels. We also noticed a similar glucose response using *C*. *pyrenoidosa* (data not shown). Taken together, the addition of glucose into medium can increase the biomass of tested *Chlorella* species similarly. In addition, the effect of other monosaccharide (fructose, galactose and xylose) supplementation on the biomass of *C*. *sorokiniana* UTEX 1230 was also revealed in this study. It was reported that *C*. *sorokiniana* ATCC-2252 and wild type strains was capable of utilizing xylose for enhanced growth rates when grown in the light, but not when grown heterotrophically in the dark [33]. D-glucose induced *C*. *sorokiniana* UTEX 1602 cells (the alga was heterotrophically grown on D-glucose and then harvested as D-glucose induced cells) exhibited a remarkably increased D-xylose uptake rate, which suggested the existence of an inducible D-xylose transportation system and a related metabolic pathway in microalga [34]. Glucose is the primary metabolic fuel for humans and other animals. It is more stable than other monosaccharides and is less susceptible to the formation of nonspecific glycoconjugates. It is for this reason that pathways for rapid conversion from other monosaccharides have been highly conserved) among many animals [35, 36]. In this study, it is demonstrated that glucose is the primary monosaccharides being used for *C*. *sorokiniana* UTEX 1230.

### The effect of glucose supplementation on lipid biosynthesis in *C*. *sorokiniana* UTEX 1230

When there is an oversupply of carbohydrate, the excess carbohydrate is converted to lipid. This involves the synthesis of fatty acids from acetyl-CoA and the esterification of fatty acids in the production of triglycerides, a process called lipogenesis. In this study, it is demonstrated that lipid yield increased with the supplementation of glucose, although the lipid content per dry weight of biomass decreased slightly at low concentration of glucose (<4g/L), while increased dramatically at high concentration glucose (≥4g/L). This fact indicated that oversupplied glucose was preferentially converted to lipid, microscopic observation at high cell density revealed that the cells of *C*. *sorokiniana* UTEX 1230 became larger at low cell density in high-magnification microscopic image [37].

### The effect of glucose supplementation on protein biosynthesis in *C*. *sorokiniana* UTEX 1230

In glucose-supplemented medium, the relative protein abundance per unit culture volume increased slightly at lower concentrations (1 g/L and 4g/L), but at similar level at higher concentration (8 g/L and 16 g/L), while the relative protein abundance per unit dry weight of biomass decreased under all concentrations examined. We assume that part of the energy supposed to be used for protein biosynthesis was switched into lipid metabolism. Both glucose transformation and decreased protein biosynthesis contributed to the increase of lipid content. From this perspective, glucose supplementation can provide a stress for the induction of lipid production. As is well known, depletion of nitrogen or phosphorus from the culture medium can increase *C*. *sorokiniana* UTEX 1230 lipid content; however, such depletion can also dramatically decrease biomass. Li et al. [38] found that depletion of nitrogen from *C*. *protothecoides* culture medium doubled lipid content while significantly decreasing biomass, with the net effect being slightly increased lipid yield. Shen et al. [39] reported that nitrogen starvation in glucose-supplemented (12 g/L) medium resulted in an 89% increase in fatty acid methyl ester in *C*. *vulgaris*, whereas no such effect was observed for a phosphorus starvation treatment. It should be pointed out that depletion of components such as nitrogen or phosphorus from culture medium, especially on an industrial scale, is a difficult task. Supplementation with glucose, as reported in this study, is operationally much easier at least in laboratories. However, in large scale cultivation process, the increased cost and contamination rate will be a problem for its applications. Usually, the concentration of total protein was used as a key feature for nutritional value, thus measures taken for the decrease of protein concentration are not preferable in practical development.

In this study, four monosaccharides (glucose, fructose, galactose and xylose) were individually supplemented into medium as carbon sources for the cultivation of *C*. *sorokiniana* UTEX 1230. It was found that supplementation with glucose increased OD_750_, biomass and lipid yield but decreased protein abundance per unit dry weight of biomass. A low concentration of glucose (<4 g/L) simultaneously promoted the biosynthesis of chlorophylls and protein abundance per unit culture volume, but decreased the lipid content per unit dry weight of biomass. Higher glucose concentrations (≥4 g/L) decreased the biosynthesis of chlorophylls and protein abundance per unit culture volume, but increased the lipid content per unit dry weight of biomass. In glucose supplemented scenario, *C*. *sorokiniana* UTEX 1230 growth was light-independent. Our findings represent basic experimental data on the effect of monosaccharides and can serve as the basis for a robust cultivation system in laboratory to increase biomass and lipid yield.

## Supporting information

S1 Data RAW data for Fig 2. (XLSX)

S2 Data RAW data for Fig 3. (XLSX)

S3 Data RAW data for Fig 4. (XLSX)

S4 Data RAW data for Fig 5. (XLSX)

S5 Data RAW data for Fig 6. (XLSX)

S6 Data RAW data for Fig 7B. (XLSX)

S7 Data RAW data for Fig 7C. (XLSX)

S8 Data RAW data for S2 Fig. (XLSX)

S9 Data RAW data for S3 Fig. (XLSX)

S10 Data RAW data for S4 Fig. (XLSX)

S11 Data RAW data for S5 Fig. (XLSX)

S12 Data RAW data for S6 Fig. (XLSX)

S13 Data RAW data for S7 Fig. (XLSX)

S1 Fig Cellular alterations of *C*. *sorokiniana* UTEX 1230 grown in monosaccharide-supplemented medium. Chlorella cells cultivated for 7 days under different concentration of monosaccharide were observed under light microscope with the aid of hemocytometer. (a) Dark condition; (b) Light condition.
Bar = 100μm.
(TIF)

S2 Fig Cell number alteration of *C*. *sorokiniana* UTEX 1230 grown in monosaccharide-supplemented medium. Cell number of 7-day batch culture supplemented with glucose (a), fructose (b), galactose (c) and xylose (d) were counted using hemocytometer, black bars indicate the cells grown under dark, white bars indicate the cells grown under light. Data shown as mean +/-SD, n = 3.
(TIF)

S3 Fig Lipid content per unit dry weight of *C*. *sorokiniana* UTEX 1230 in glucose-supplemented medium. Lipid production for *C*. *sorokiniana* UTEX 1230 cells collected from glucose-supplemented medium at 7 days was determined by SPV method as described in the method section. The lipid contents per unit dry weight (μg/mg) were calculated. The bar graph was drawn with the glucose concentration as abscissa and the lipid content per unit dry weight as ordinate. Data shown as mean +/-SD, n = 3.
Black bar: dark condition; white bar: light condition.
(TIF)

S4 Fig Correlation between OD_750_ and cell number. The OD_750_ and cell number of *C*. *sorokiniana* UTEX 1230 cells from day 0 to day 7 were measured by spectrophotometer and counted using hemocytometer, respectively. The left and right Y-axis are cell number and OD_750,_ respectively. Data shown as mean +/-SD, n = 3.
(TIF)

S5 Fig Relative protein abundances per cell of *C*. *sorokiniana* UTEX 1230 cultured in glucose-supplemented medium. *C*. *sorokiniana* UTEX 1230 cells cultured in glucose-supplemented medium were harvested. The extracted total protein was separated by SDS-PAGE (10%) and stained with Coomassie brilliant blue (Fig 7A). Signal intensities were extracted by ImageJ software and divided by cell number. Data shown as mean +/-SD, n = 3.
(TIF)

S6 Fig The standard curve of protein concentration determined by Bradford method. Different concentration of Bovine serum albumin (BSA) were mixed with Coomassie Bright Blue G-250 solution (100 mL solution containing: 0.01 g Coomassie Bright Blue G-250, 5 mL 90% ethanol and 10 mL 85% phosphoric acid) and used as standard samples, the optical density at 595 nm were measured by spectrophotometer (7200 Unico, Shanghai, China). The X-axis is BSA concentration, the Y-axis is OD_595_, the linear regression equation was generated by Excel software. Data shown as mean +/-SD, n = 3.
(TIF)

S7 Fig Correlation between CBB and Bradford method determined protein concentration. Total proteins from *C*. *sorokiniana* UTEX 1230 cultures supplemented with 0 g/L and 4 g/L glucose at day 0, 1, 3, 5 and 7 were extracted. The protein concentration was determined by CBB and Bradford method in parallel. In CBB method, the total proteins were separated by SDS-PAGE and stained with Coomassie Bight Blue R-250 (a). The intensities of stained gel were collected by a Mini Chemiluminescent Imaging system and Lane 1D Analysis software (Sage Creation Science Co., Ltd., Beijing, China). In Bradford method, the optical density at 595 nm was measured by spectrophotometer (7200 Unico, Shanghai, China), the protein concentrations were calculated based on the standard curve (S6 Fig). Normalized signals were used to compare the relative intensities determined by CBB and Bradford method (b). To normalize the data, the sum of signal intensities collected by CBB and Bradford method were set to an equal amount of value, and the relative signals for each sample was calculated respectively. Data shown as mean +/-SD, n = 3.
Blue bars represent the relative intensities determined by CBB. White bars represent the relative intensities determined Bradford method.
(TIF)
