# Supplementary figures and images for: Characterization of Chlorella sorokiniana growth properties in monosaccharide-supplemented batch culture
Source: PLoS One. 2018 Jul 3;13(7):e0199873. doi: 10.1371/journal.pone.0199873 (PMC6029798; doi:10.1371/journal.pone.0199873)

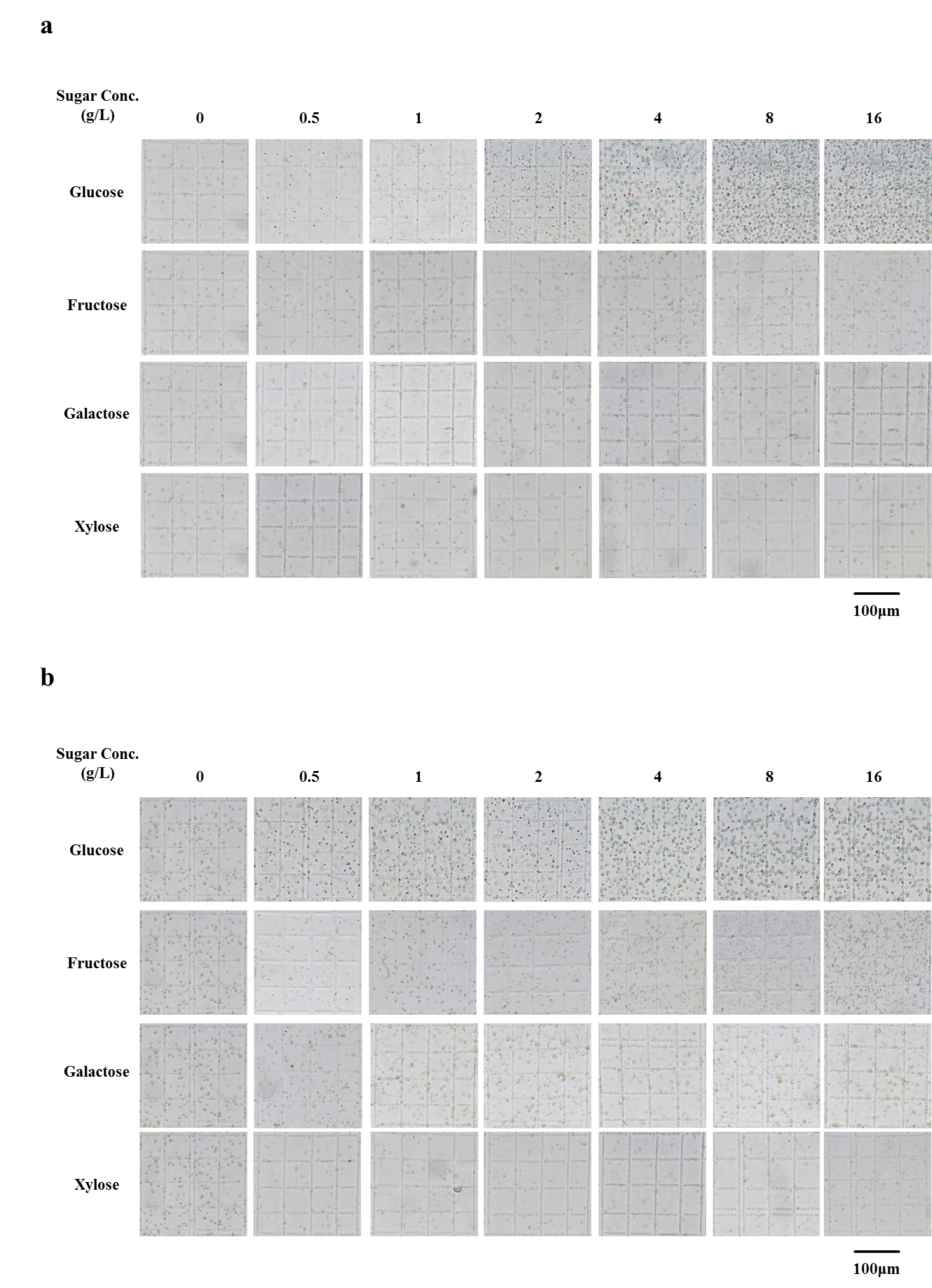

Supplement: S1 Fig — Chlorella cells cultivated for 7 days under different concentration of monosaccharide were observed under light microscope with the aid of hemocytometer. (a) Dark condition; (b) Light condition. Bar = 100μm. (TIF) [file pone.0199873.s014.tif]

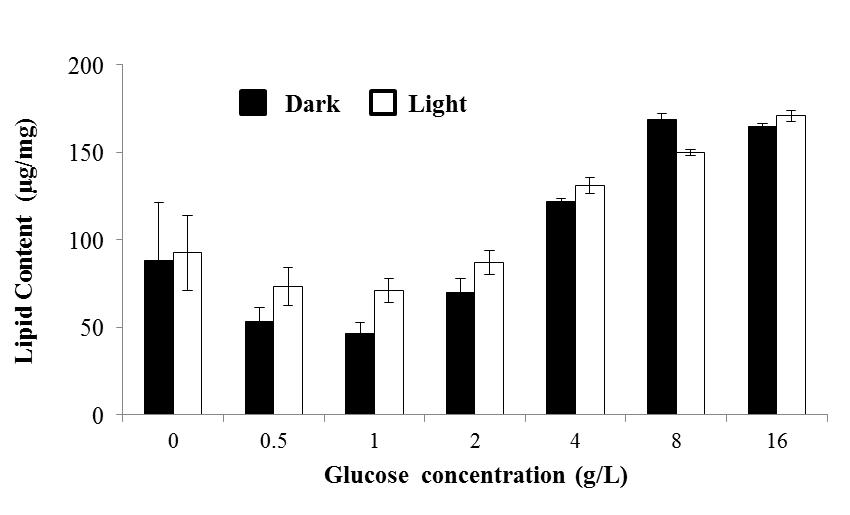

Supplement: S2 Fig — Cell number of 7-day batch culture supplemented with glucose (a), fructose (b), galactose (c) and xylose (d) were counted using hemocytometer, black bars indicate the cells grown under dark, white bars indicate the cells grown under light. Data shown as mean +/-SD, n = 3. (TIF) [file pone.0199873.s015.tif]

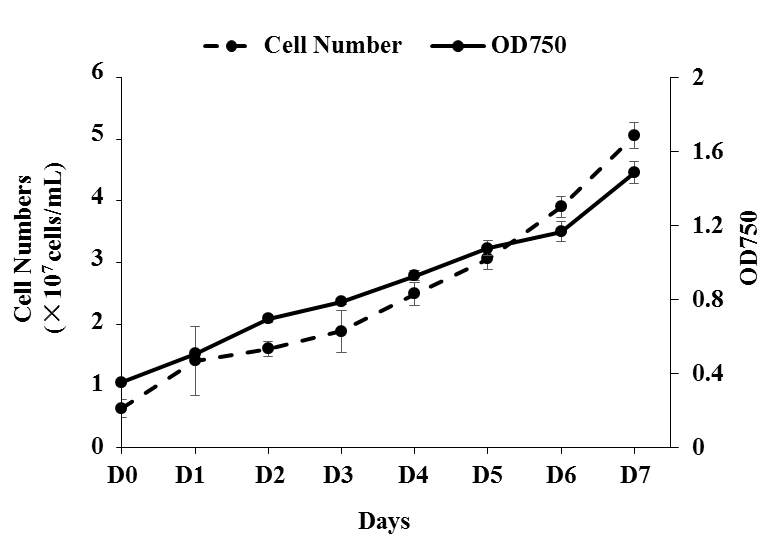

Supplement: S3 Fig — Lipid production for C. sorokiniana UTEX 1230 cells collected from glucose-supplemented medium at 7 days was determined by SPV method as described in the method section. The lipid contents per unit dry weight (μg/mg) were calculated. The bar graph was drawn with the glucose concentration as abscissa and the lipid content per unit dry weight as ordinate. Data shown as mean +/-SD, n = 3. Black bar: dark condition; white bar: light condition. (TIF) [file pone.0199873.s016.tif]

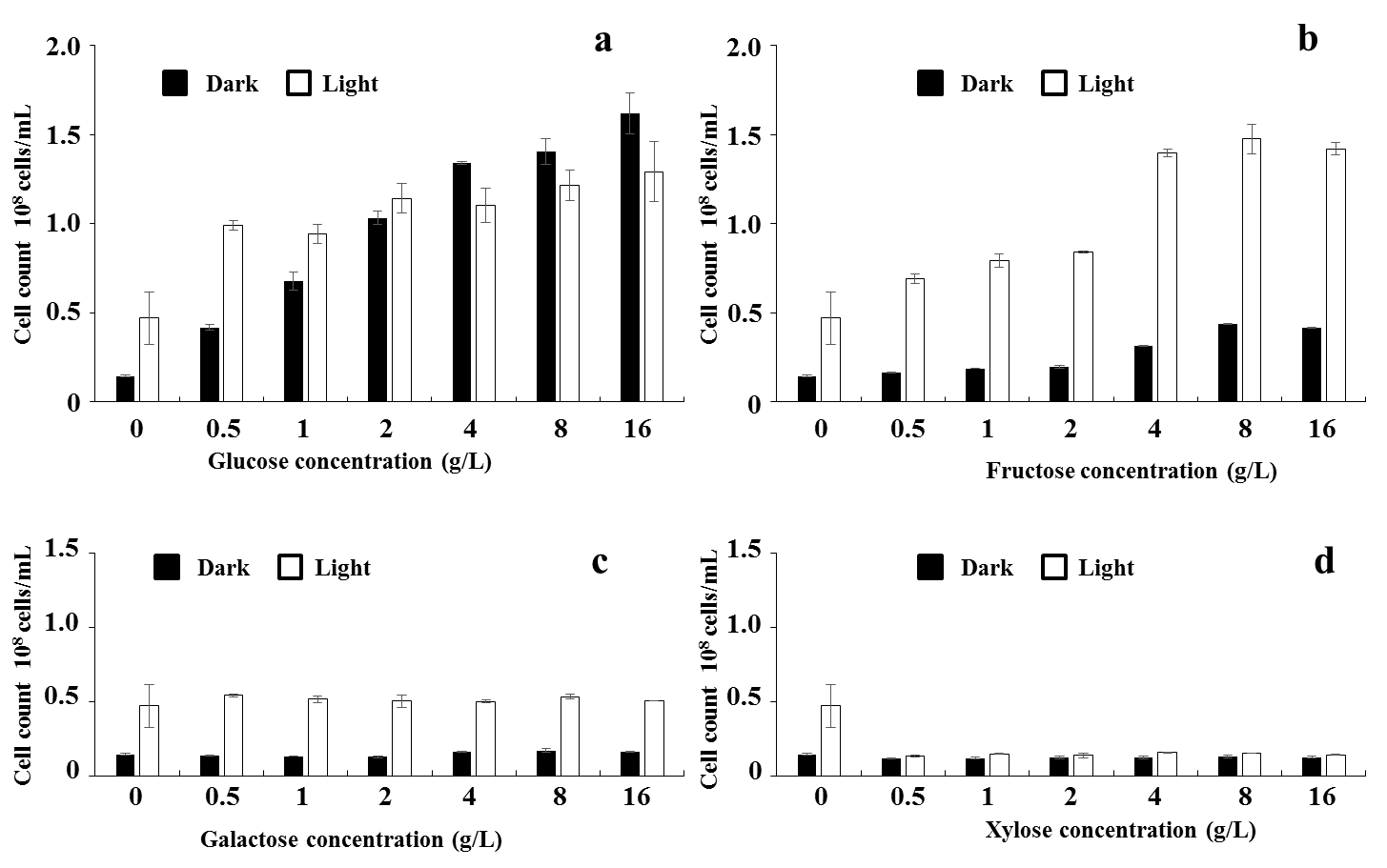

Supplement: S4 Fig — The OD750 and cell number of C. sorokiniana UTEX 1230 cells from day 0 to day 7 were measured by spectrophotometer and counted using hemocytometer, respectively. The left and right Y-axis are cell number and OD750, respectively. Data shown as mean +/-SD, n = 3. (TIF) [file pone.0199873.s017.tif]

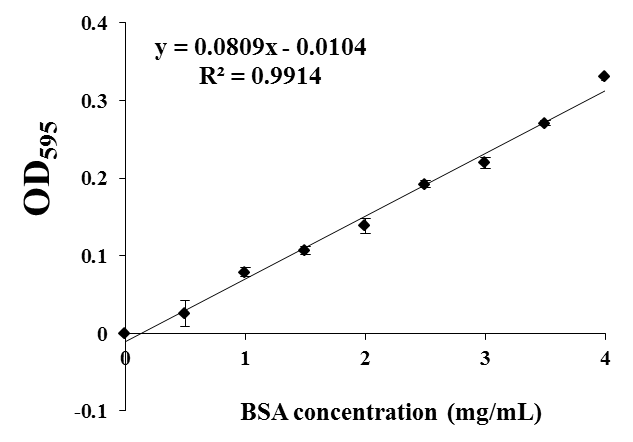

Supplement: S5 Fig — C. sorokiniana UTEX 1230 cells cultured in glucose-supplemented medium were harvested. The extracted total protein was separated by SDS-PAGE (10%) and stained with Coomassie brilliant blue (Fig 7A). Signal intensities were extracted by ImageJ software and divided by cell number. Data shown as mean +/-SD, n = 3. (TIF) [file pone.0199873.s018.tif]

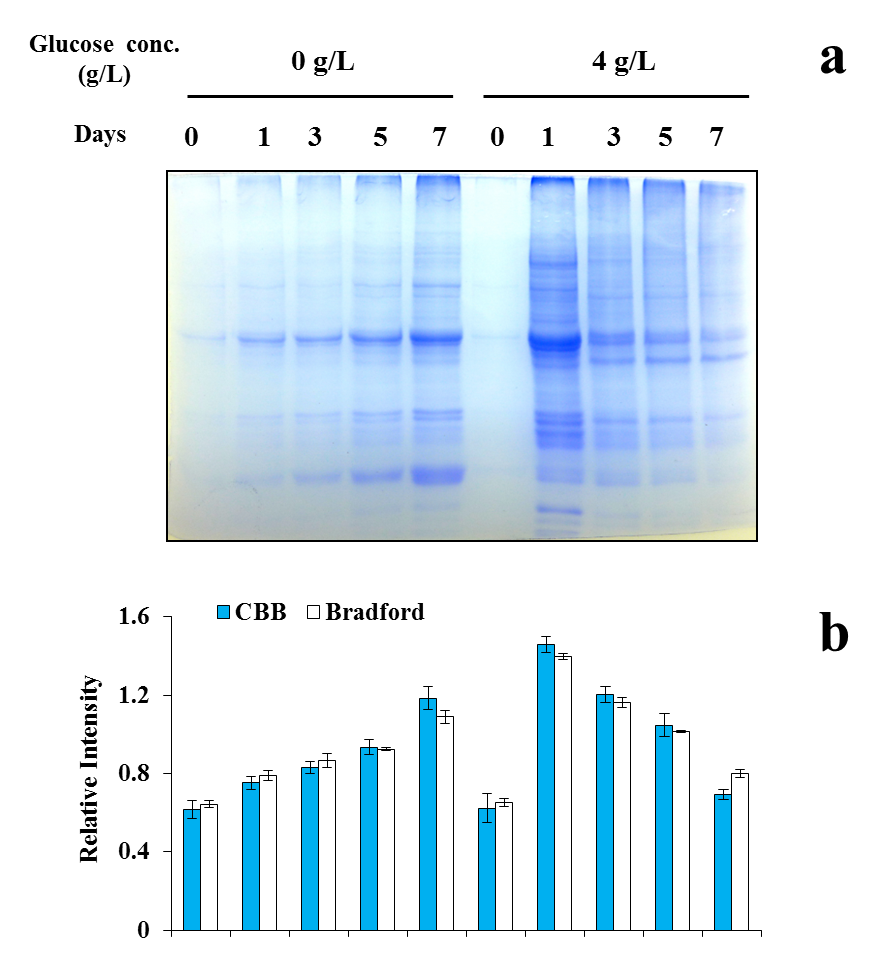

Supplement: S6 Fig — Different concentration of Bovine serum albumin (BSA) were mixed with Coomassie Bright Blue G-250 solution (100 mL solution containing: 0.01 g Coomassie Bright Blue G-250, 5 mL 90% ethanol and 10 mL 85% phosphoric acid) and used as standard samples, the optical density at 595 nm were measured by spectrophotometer (7200 Unico, Shanghai, China). The X-axis is BSA concentration, the Y-axis is OD595, the linear regression equation was generated by Excel software. Data shown as mean +/-SD, n = 3. (TIF) [file pone.0199873.s019.tif]

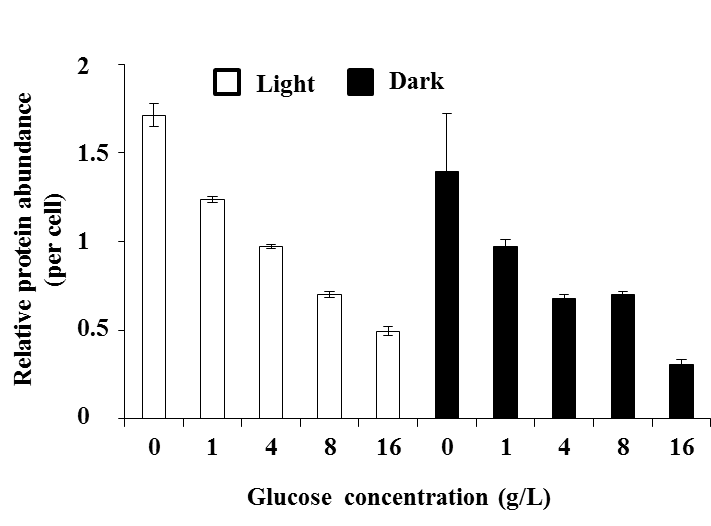

Supplement: S7 Fig — Total proteins from C. sorokiniana UTEX 1230 cultures supplemented with 0 g/L and 4 g/L glucose at day 0, 1, 3, 5 and 7 were extracted. The protein concentration was determined by CBB and Bradford method in parallel. In CBB method, the total proteins were separated by SDS-PAGE and stained with Coomassie Bight Blue R-250 (a). The intensities of stained gel were collected by a Mini Chemiluminescent Imaging system and Lane 1D Analysis software (Sage Creation Science Co., Ltd., Beijing, China). In Bradford method, the optical density at 595 nm was measured by spectrophotometer (7200 Unico, Shanghai, China), the protein concentrations were calculated based on the standard curve (S6 Fig). Normalized signals were used to compare the relative intensities determined by CBB and Bradford method (b). To normalize the data, the sum of signal intensities collected by CBB and Bradford method were set to an equal amount of value, and the relative signals for each sample was calculated respectively. Data shown as mean +/-SD, n = 3. Blue bars represent the relative intensities determined by CBB. White bars represent the relative intensities determined Bradford method. (TIF) [file pone.0199873.s020.tif]
